# Stage-Specific Survival Rate of Breast Cancer Patients in Northern Thailand in Accordance with Two Different Staging Systems

**DOI:** 10.31557/APJCP.2019.20.9.2699

**Published:** 2019

**Authors:** Imjai Chitapanarux, Patumrat Sripan, Areewan Somwangprasert, Chaiyut Charoentum, Wimrak Onchan, Kirati Watcharachan, Panchaporn Wongmaneerung, Pailin Kongmebhol, Bongkot Jia-Mahasap, Lalita Huntrakul

**Affiliations:** 1 *Division of Radiation Oncology,*; 2 *Northern Thai Research Group of Radiation Oncology (NTRG-RO), *; 4 *Department of Surgery,*; 5 *Oncology Unit, Department of Medicine, *; 6 *Department of Radiology, Faculty of Medicine, *; 3 *Chiang Mai Cancer Registry, Maharaj Nakorn Chiang Mai Hospital, Faculty of Medicine,Chiang Mai University, Chiang Mai, Thailand.*

**Keywords:** Breast cancer, overall survival, AJCC staging, SEER staging

## Abstract

**Objective:**

This study was attempted to investigate overall survival by stage at diagnosis in female breast cancer patients in Northern Thailand by using 2 different staging systems; namely, American Joint Committee on Cancer (AJCC) Tumor (T), Nodal (N) and Metastatic (M) staging system and Surveillance Epidemiology and End Results (SEER) summary staging system.

**Methods:**

We studies female breast cancer patients whose data were registered in Chiang Mai cancer registries between January 2006 and December 2015. Data were recorded in SEER summary staging system. The TNM AJCC staging was searched in the medical records.

**Results:**

A total of 3,873 female breast cancer patients were diagnosed from 2006-2015. All data sets were recorded in SEER summary stage 2000. Early stage was the most prevalent stage at the time of diagnosis (58%), followed by loco-regional advanced stage (32%), and metastatic breast cancer (10%). The 5-year overall survival rate of early, loco-regional advanced, and metastatic stages were 85.3%, 66.4%, and 26.2%, respectively. After examining the medical records, we excluded patients who had no data on T, N, and M in their records. Finally, only 3,251 patients were analyzed for AJCC stage-specific survival. The 5-year overall survival rate in stages I, II, III, and IV were 94.4%, 85.0%, 56.6%, and 28.3%, respectively.

**Conclusion:**

Comparing to more stable economic countries, the 5-year overall survival rate for a specific stage of breast cancer in Northern Thailand was slightly lower in early stage and stage I-II in accordance with AJCC, but much lower in loco-regional stage and stage III with respect to AJCC. Nevertheless, it was similar in metastatic stage and stage IV according to AJCC.

## Introduction

Breast cancer is the most commonly diagnosed cancer in Thai women, with the age-standardized incidence rate (ASR) of 28.5 per 100,000 people in Thailand and 27.4 per 100,000 people in Chiang Mai (Imsamran et al., 2015). Most cancer registries in Thailand do not routinely record staging data of Tumor (T), Nodal (N), and Metastatic (M). TNM staging is used frequently by the clinicians (Kwan et al., 2012) and also recommended by American Joint Committee on Cancer (AJCC).

Staging was commonly reported in Thailand using Surveillance Epidemiology and End Results (SEER) registries (Kwan et al., 2012). Chiang Mai cancer registry had previously collected data on staging of breast cancer for Global Burden of Cancer Study (GLOBOCAN) using SEER Summary Stage-2000, which categorized the extent of the disease into following stages : in situ, localized, regional, and distant metastasis (Young et al., 2001).

Nevertheless, all clinicians in our center recorded and used TNM staging for the diagnosis and management of. The objective of the current study was to analyze the overall survival (OS) of breast cancer in stage of distribution based on data reported by our cancer registry from 2006 to 2015.For data collection, we used SEER Summary Stage-2000 as well as TNM staging by AJCC 6^th^ and 7^th^ edition (Edge, 2002; Edge et al., 2011).

## Materials and Methods

We conducted a retrospective cohort study by examining the data on female patients with breast cancer who were diagnosed from January 2006 to December 2015. Patients’ data (ICD10 code C50) were extracted from Chiang Mai Cancer Registry and medical records. The alive or deceased status of the patients and the date of death were obtained by retrieving the mortality data from the National Registration Department, Ministry of Interior. In this study, we analyzed the stage of the disease at the time of the diagnosis using two systems; namely, SEER Summary Stage-2000 (Young et al., 2001) and TNM staging presented by AJCC staging system ( the 6^th^ and 7^th^ edition) (Edge, 2002; Edge et al., 2011). Patients who had incomplete T, N, and, M records were excluded from the survival analysis according to AJCC staging.

Overall survival was defined as time from diagnosis of breast cancer to death of patient due to any cause. Patients were censored at date of last follow up. Patients’ medical information such as TNM stage, type of treatment (i.e. chemotherapy or radiotherapy), and follow up data were obtained from their medical records. Three prognostic indicators; namely, estrogen and progesterone receptors as well as HER2 status were used to classify the subtype of breast cancer in patients. Statistical analyses were run using Stata version 11 (StataCorp LP, College Station, TX, USA). Frequency tables were applied for univariate analysis for overall survival were based on the Kaplan Meier method, where stratum-specific outcomes were compared using log rank statistics. The concordance of five-year overall survival of all patients, staged by two different staging systems, was also evaluated using log rank statistics. The variables found significant in the univariate analysis were included in the multivariate model. The final survival analysis model was adjusted for potential confounding variables. All of the employed statistical tests were two-sided, and P< 0.05 was regarded to discern any statistically significant differences in outcomes.

## Results

**Figure 1:**
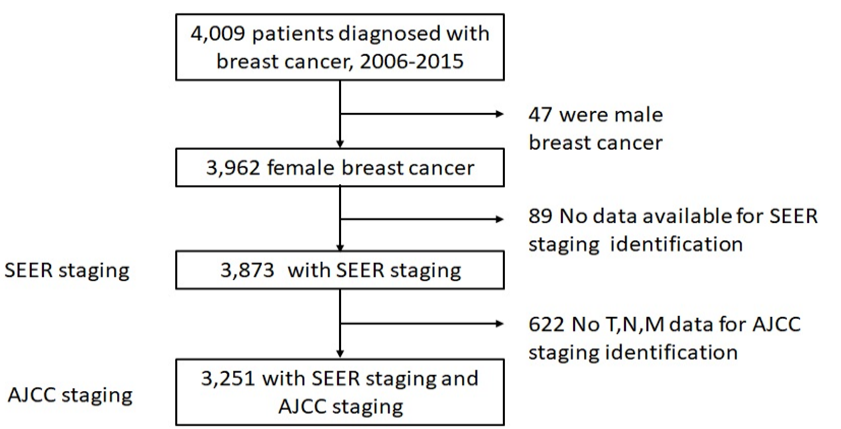
Flow Diagram of Studies Patients

Among 3,962 female breast cancer patients, the number of cases with complete data suitable for the stage specific survival analysis by using SEER summary stage-2000 and AJCC staging systems were 3,873 and 3,251 cases, respectively. Figure 1 shows the flow diagram of studied patients. 

The most prevalent age of diagnosis was 40-60 years old in our study. The median age of the patients was 52 years old (IQR : 45-59) in both staging systems. Baseline characteristics of the patients staged by both systems are shown in Table 1. In both staging systems, the percentage of patients aged between 40 and 60 was higher in all stages compared to other age groups. The most common subtype of breast cancer was luminal B in all stages in both staging systems. According to AJCC staging system, 40% of patients (N=445) had unknown hormone receptor and HER-2 status of the tumor. Over half of the patients in this cohort study received chemotherapy following national reimbursement scheme even though they were in early stage and had AJCC stage I disease (i.e T1c tumor > 10 mm). The percentage of patients who received radiotherapy increased with the severity of disease stage at the time of diagnosis; that is, 45% and 72% for localized and regional stage and 35%, 55%, and 76% for AJCC stage I, II, and III, respectively. Approximately half of the patients with metastatic stage according to SEER summary stage-2000 or AJCC stage IV also received radiotherapy for palliative intent (44% versus 48%) such as palliative to brain, bone metastasis, and superior vena cava syndrome . 

**Figure 2:**
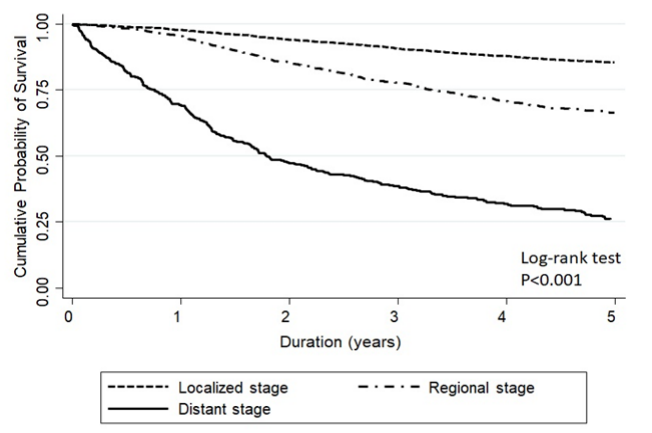
Five-Year Overall Survival of Patients Staged by SEER Summary Stage-2000 System

**Figure 3:**
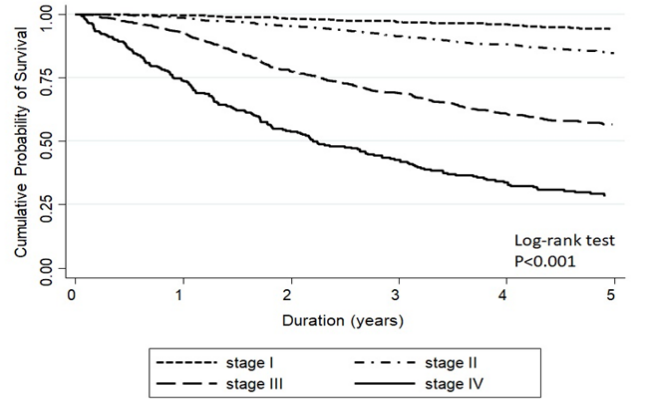
Five-Year Overall Survival of Patients Staged by AJCC Staging System

**Figure 4:**
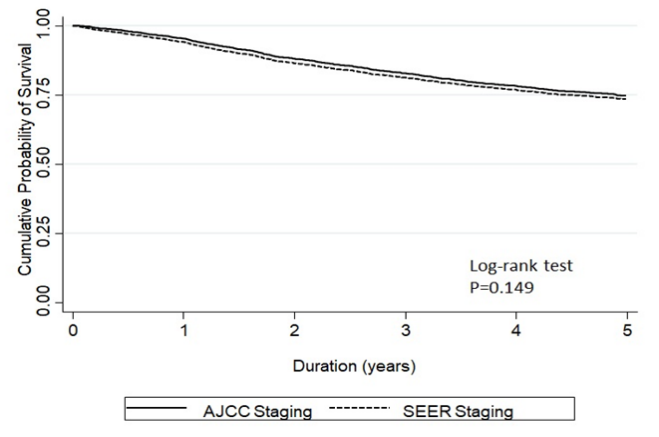
Concordance of Five-Year Overall Survival of All Patients Staged by Two Staging Systems

Figure 2 and 3 show the Kaplan Meier curve of stage-specific survival of patients staged by SEER summary stage-2000 and AJCC staging system, respectively. Five-year overall survival rate for all staged combined by SEER system was 74%, while the 5-year overall survival rates of early, loco-regional advanced, and metastatic stages were 85.3% (83.8-86.8), 66.4% (63.3-69.2), and 26.2% (21.7-30.9), respectively (Figure 2). Five-year overall survival rate for all stages combined by AJCC system was 75%, while 5-year overall survival rates in stages I, II, III, and IV were 94.4% (92.0-96.1), 85.0% (83.0-86.8), 56.6% (53.0-60.1), and 28.3% (22.7-34.2), respectively (Figure 3). The concordance of five-year overall survival of all patients staged by two different staging system is shown in Figure 4 (p=0.149).

Table 2 and 3 show the univariable and multivariable cox proportional hazard regression analysis of 5-year overall survival in both staging systems. The log-rank test identified significant differences between 5-year overall survival rates and age, stage of the disease at the time of diagnosis, subtype of tumor, hormonal treatment, surgery, and year of diagnosis in both systems. In the multivariate analysis, it was found that age more than 60 years old, higher stage, and TNBC were associated with poor overall survival. While receiving breast conserving therapy (BCT) and modified radical mastectomy (MRM) was associated with better overall survival [hazard ratio (HR): 0.29 and 0.56, respectively, P<0.001] in both SEER stage system and AJCC staging. as well as more recent year of diagnosis (2006-2010 vs 2011-2015) and receiving hormonal therapy, HR=0.85 (p=0.014) and HR=0.70 (p<0.001), respectively, in SEER stage system and HR=0.82 (p=0.007) and HR=0.76 (p=0.003), respectively in AJCC staging. Receiving chemotherapy [HR 0.78; 95% CI, 0.66-0.93; P=0.005] was detected as another independent factor for achieving a good survival outcome in patients staged by AJCC system.

## Discussion

The stage-specific survival rates of our patients in both SEER summary stage-2000 and AJCC 6th edition were all moving in the same direction while witnessing the reduction of survival rates in later stages. The 5-year overall survival rate of localized stage was mixed between stage I and II; likewise, the 5-year overall survival rate of regional stage was mixed between stage II and III. Compared with more stable economic countries, the 5-year overall survival rate of Northern Thai breast cancer patients was lower in every stage regardless of staging system. The AJCC 7^th^ edition (Edge et al., 2011) was used in our hospital during 2010. As mentioned in a report by Kwan et al., (2012), the major change in TNM staging for breast cancers from 6th to 7^th^ edition is the new sub-division of Stage I to Stage IA and IB. Stage IB in the 7th edition includes micro-metastasis lymph nodes which were previously included in Stage IIA in 6^th^ edition. Stage grouping was also changed from 6th to 7th edition as follow: stage IB to IIA, IIB to IIA, and IIIB to IIIA. However, our study did not sub-divide stages into A or B, thus we might have the combination of stage I and II patients.

In western and more stable economic countries, breast cancer survival rates have improved significantly in past decades. SEER reported five-year survival rates of 90.6% for breast cancer patients diagnosed in 2006 (Noone et al., 2018) because of new drug development and widespread mammographic screening. The population-based Saarland Cancer Registry in Germany included invasive breast cancer patients between 2000 and 2009 and reported that the overall age standardized 5-year relative survival was 83% (Holleczek et al., 2013). A study in Japan also reported the 5-year overall survival rate of 92.7 % in 2006, which was satisfactory (Anan et al., 2015). For breast cancer patients who was diagnosed in British Columbia, Canada in 2002, the 5-year overall survival was 83% for all combination of all stages (Davidson et al., 2013). 

To focus on our neighbors in Southeast Asia, we found a Malaysian cohort study, which was conducted from 2001 to 2005. The aforementioned study reported that the overall 5-year survival rate was only 49% in that country (Abdullah et al., 2013). Tan et al., (2009) reported that the 5-year age-standardized relative survival of Singaporean women diagnosed with breast cancer was 70% from 1980 to 1999. Similarly, our study found that the 5-year overall survival rates were 74% (95%CI: 72%-75%) and 75% (95%CI: 73%-76%) for all stages of breast cancer according to SEER and AJCC staging systems, respectively, which is similar to rates reported in our neighbors, but still lower than those reported in more stable economic countries s of. 

However, when we analyzed the stage-specific survival, we found that the 5-year survival was likely stratified by the stage of breast cancer. The survival rates of Thai women were comparable to those of stable economic countries. We found that the 5-year overall survival of localized and metastatic stages was slightly lower while much lower in regional stage when compared with the results of a study conducted in German, indicating that ... (Holleczek et al., 2013). In addition, with respect to AJCC staging, the 5-year overall survival of stages I, II, and IV in our cohort was slightly lower while much lower in stage III in comparison with the results of studies conducted in the US and Japan, showing that… (Edge et al., 2011; Anan et al., 2015). However, in a Canadian report, similar findings were reported, which demonstrated that (Davidson et al., 2013). 

Our findings are consistent with those of other research conducted in Southeast Asian countries. The results of a study conducted based on data retrieved from Singapore-Malaysia hospital-based breast cancer registry showed that the 5-year overall survival was 82.5% in patients with TNM stage 0-II cancer, and 30.2% in patients at late stages (Pathy et al., 2011). Five-year age-standardized relative survival by the stage of cancer in relation to time since diagnosis in a Singaporean study (Tan et al., 2009) was 87%, 60%, and 15% for localized, regional, and distant metastatic stages, respectively. Nevertheless, they analyzed patients in the earlier year of diagnosis than our study where the lack of novel treatments might affect their outcomes. Table 4 demonstrates the results of comparison between our study and other countries in terms of the 5-year survival outcomes .

In Thailand, there is limitation of screening mammography. The recent breast cancer screening guideline presented by our National Cancer Institute recommendes mass, selective, or opportunistic screening. This can explain why our study showed a stage distribution more weighted to stages II-III than stage I. Most Thai patients are covered under Medical Welfare Scheme (MWFS). Patients can access public health services in public hospitals and private hospital affiliated with the National Health Security Office (NHSO). The Thai government Universal Coverage Health Scheme, MWFS, offers three cornerstones of breast cancer treatment; that is, surgery, chemotherapy, and radiotherapy. However, NHSO restricts access to certain critical medical treatments, such as novel dose-dense chemotherapy treatments, anti-hormonal therapy or ovarian suppression, and anti-HER2 treatment. The most widely used adjuvant chemotherapy regimens were FAC (5-Fluorouracil, adriamycin, cyclophosphamide), AC (adriamycin, cyclophosphamide), and CMF (cyclophosphamide, methotrexate, and fluorouracil). In Thailand, Taxane was approved in 2007, which restricted indication in the sequential adjuvant treatment after AC for high-risk node-positive breast cancer by 2007 St Gallen expert consensus criteria. Afterwards, non-steroidal aromatase inhibitor (letrozole only) was approved by NHSO in 2009 to be used nationwide for adjuvant therapy for node-positive hormone-receptor positive postmenopausal breast cancer patients. Most patients in this analysis did not received adjuvant trastuzumab since this agent was only recently approved in 2015 for node-positive Her 2-positive breast cancer patients. 

**Table 1:** Patients' Baseline Characteristics in Accordance with 2 Systems of Staging

Variables	SEER Summary Stage 2000 (N=3,873)			AJCC staging (N= 3,251)			
	Early stage n (%)	Loco-regional stage n (%)	Metastatic stage n (%)	stage I n (%)	stage II n (%)	stage III n (%)	stage IV n (%)
Number of patients	2,258 (58)	1,219 (32)	396 (10)	589 (18)	1,564 (48)	836 (26)	262 (8)
Median age (year)	51 (45-59)	52 (45-58)	54 (46-62)	53 (46-60)	51 (45-59)	52 (45-58)	54 (48-62)
Age (year)							
<40	269 (12)	144 (12)	33 (8)	60 (10)	201 (13)	86 (10)	16 (6)
40-44	273 (12)	144 (12)	43 (11)	68 (12)	182 (11)	112 (14)	30 (11)
45-49	413 (19)	215 (18)	63 (16)	97 (16)	282 (18)	164 (20)	42 (16)
50-54	433 (19)	238 (20)	69 (17)	116 (20)	307 (20)	151 (18)	51 (19)
55-59	326 (14)	202 (17)	70 (18)	95 (16)	245 (16)	128 (15)	44 (17)
> 60	544 (24)	276 (23)	118(30)	153 (26)	343 (22)	194 (23)	84 (31)
Subtype							
Luminal A	575 (26)	275 (22)	64 (16)	195 (33)	421 (27)	147 (18)	43 (17)
Luminal B	637 (28)	389 (32)	113 (29)	202 (35)	458 (29)	274 (33)	87 (33)
Her-2	321 (14)	231 (19)	76 (19)	77 (13)	247 (16)	172 (20)	69 (26)
Triple negative	268 (12)	154 (13)	39 (10)	49 (8)	224 (14)	120 (14)	21 (8)
Unknown	457 (20)	170 (14)	104 (26)	66 (11)	214 (14)	123 (15)	42 (16)
Surgery							
No surgery	215 (10)	122 (10)	169 (43)	35 (6)	89 (6)	91 (11)	105 (39)
BCT	519 (23)	96 (8)	31 (8)	226 (39)	271 (17)	28 (3)	16 (6)
MRM	1,524 (67)	1,001 (82)	196 (49)	328 (56)	1,200 (77)	716 (86)	146 (55)
Chemotherapy							
No	785 (35)	237 (19)	158 (40)	276 (47)	317 (20)	150 (18)	76 (29)
Yes	1,473 (65)	982 (81)	238 (60)	313 (53)	1,247 (80)	686 (82)	186 (71)
Radiotherapy							
No	1,237 (55)	338 (28)	220 (56)	385 (65)	697 (45)	203 (24)	137 (52)
Yes	1,021 (45)	881 (72)	176 (44)	204 (35)	867 (55)	633 (76)	125 (48)
Hormonal therapy							
No	1,120 (50)	707 (58)	265 (67)	218 (37)	784 (50)	507 (61)	156 (60)
Yes	1,138 (50)	512 (42)	131 (33)	371 (63)	780 (50)	329 (39)	106 (40)
Year of diagnosis							
2006-2010	1,285 (57)	410 (34)	212 (54)	269 (46)	786 (50)	366 (44)	136 (51)
2011-2015	973 (43)	809 (66)	184 (46)	320 (54)	774 (50)	469 (56)	131 (49)

BCT, Breast conserving therapy; MRM, Modified radical mastectomy

**Table 2:** Univariable Cox Proportional Hazard Regression Analysis of 5-Year Overall Survival in Both Staging Systems

Covariates	SEER Summary Stage 2000 (N=3,873)			AJCC staging (N=3,251)		
	Hazard Ratio (HR)	95% CI	†P-value	Hazard Ratio (HR)	95% CI	†P-value
Age (years)						
<40	1			1		
40-44	0.96	0.75- 1.22	0.735	1.06	0.80-1.40	0.676
45-49	0.99	0.79-1.23	0.905	1.08	0.83-1.39	0.572
50-54	0.95	0.76-1.18	0.64	1.05	0.81-1.35	0.726
55-59	1.05	0.84-1.33	0.655	1.1	0.85-1.43	0.471
> 60	1.54	1.26-1.89	<0.001	1.57	1.24-1.99	<0.001
Stage						
I	-	-		1		
II	-	-		2.47	1.82-3.36	<0.001
III	-	-		7.87	5.81-10.66	<0.001
IV	-	-		18.9	13.73-26.02	<0.001
Early stage	1			-	-	-
Loco-regional stage	2.34	2.05-2.68	<0.001	-	-	-
Metastatic stage	8.48	7.31-9.84	<0.001	-	-	-
Subtype						
Luminal A	1			1		
Luminal B	1.18	0.98-1.41	0.076	1.18	0.97-1.44	0.092
Her-2 positive	1.81	1.50-2.19	<0.001	1.91	1.56 -2.35	<0.001
Triple negative	1.91	1.56-2.34	<0.001	1.91	1.53-2.38	<0.001
Unknown	1.78	1.49-2.13	<0.001	1.8	1.45-2.22	<0.001
Surgery						
No surgery	1			1		
BCT	0.16	0.12-0.20	<0.001	0.14	0.10-0.18	<0.001
MRM	0.39	0.34-0.44	<0.001	0.35	0.30-0.41	<0.001
Chemotherapy						
No	1			1		
Yes	0.97	0.80-1.03	0.124	0.92	0.79-1.06	0.246
Radiotherapy						
No	1			1		
Yes	1.05	0.94-1.18	0.371	1.15	1.00-1.31	0.042
Hormonal therapy						
No	1			1		
Yes	0.53	0.47-0.60	<0.001	0.54	0.48-0.62	<0.001
Year of diagnosis						
2006-2010	1			1		
2011-2012	0.87	0.79-1.02	0.084	0.86	0.75-0.99	0.035

†P-value from partial likelihood ratio tests; BCT, Breast conserving therapy, MRM, Modified radical mastectomy

**Table 3:** Multivariable Cox Proportional Hazard Regression Analysis of 5-Year Overall Survival in Both Staging Systems

Covariates	SEER Summary Stage 2000 (N=3,873)			AJCC staging (N=3,251)		
	Adjusted Hazard Ratio (aHR)	95% CI	†P-value	Adjusted Hazard Ratio (aHR)	95% CI	†P-value
Age (years)						
<40	1			1		
40-44	0.88	0.69-1.13	0.314	0.86	0.65-1.14	0.29
45-49	0.95	0.76-1.19	0.643	0.9	0.70-1.16	0.411
50-54	0.9	0.72-1.12	0.334	0.92	0.71-1.19	0.515
55-59	0.91	0.72-1.15	0.45	0.94	0.72-1.23	0.647
> 60	1.42	1.16-1.75	0.001	1.29	1.01-1.64	0.041
Stage						
I	-	-	-	1		
II	-	-	-	2.36	1.73-3.23	<0.001
III	-	-	-	7.37	5.38-10.10	<0.001
IV	-	-	-	15.03	10.78-20.97	<0.001
Early stage	1			-	-	-
Loco-regional stage	2.28	1.99-2.62	<0.001	-	-	-
Metastatic stage	6.67	5.88-8.02	<0.001	-	-	-
Subtype						
Luminal A	1			1		
Luminal B	1.03	0.86-1.23	0.778	0.97	0.80-1.19	0.783
Her-2 positive	1.13	0.90-1.40	0.286	1.12	0.88-1.42	0.36
Triple negative	1.4	1.11-1.77	0.004	1.38	1.07-1.79	0.013
Unknown	1.05	0.86-1.29	0.633	0.99	0.77-1.26	0.924
Surgery						
No surgery	1			1		
BCT	0.29	0.22-0.38		0.37	0.27-0.51	<0.001
MRM	0.56	0.48-0.65		0.55	0.45-0.66	<0.001
Chemotherapy						
No				1		
Yes				0.78	0.66-0.93	0.005
Hormonal therapy						
No	1			1		
Yes	0.7	0.60-0.83	<0.001	0.76	0.63-0.91	0.003
Year of diagnosis						
2006-2010	1					
2011-2012	0.85	0.75-0.97	0.014	0.82	0.71-0.95	0.007

†P-value from partial likelihood ratio tests; BCT, Breast conserving therapy; MRM, Modified radical mastectomy

**Table 4:** Comparison of 5-Year Overall Survival/ Relative Survival Accorrding to Two Different Staging Systems

Stage	SEER Data† (6)	German† (7)	Japan (8)	Canadian (9)	Malaysia (10)	Singaporea (11)	Singapore/Malaysia (12)	Our study
All stage combined	90.6	83	92.7	83	49	70	-	74%‡ 75%§
I	100	-	95	90	-	-	0-II 82.5	94.4
II	93	-	90	82	-	-		85
III	72	-	79	60	-	-	III-IV 30.2	56.6
IV	22	-	52	18	-	-		28.3
Localized	-	96.8	-	-	-	90	-	85.3
Regional	-	79.2	-	-	-	68	-	66.4
Distant metastasis	-	21.8	-	-	-	28	-	26.2

†, Relative survival; ‡SEER, staging 2000; §, AJCC staging

Other issues that may lead to lower survival outcome of patients with stage III disease in this study included the inadequacy of health care providers, therapeutic agents and facilities, and limited geographical location of accessible health care service centers. Patients with operable locally advanced stage III mostly received systemic adjuvant therapy, which was similar to therapy suggested for stage II disease. Sequential adjuvant taxanes mainly paclitaxel after AC was also restricted and given in high risk node-positive breast cancer patients by 2007 St Gallen expert consensus criteria. For patients with inoperable, non-inflammatory, stage III locally advanced breast cancer at presentation, the initial available chemotherapy regimen of the national reimbursement protocol is the use of either CMF, FAC, or AC without taxanes. Trastuzumab for HER-2 positive and aromatase inhibitor for hormonal positive are generally restricted in the adjuvant setting for patients treated under NHSO. These were great barriers against improving survival outcome of breast cancer patients in this area to the satisfactory level. We have only 2 radiotherapy centers in Northern Thailand and 10 radiation oncologists to serve more than 6 million populations. 

Post therapy surveillance and follow up for patients with stage I – III disease are mainly based on the performance of regular medical history and physical examination and optimal for annual mammography . 

In conclusion, according to the data of this study, the 5-year stage-specific survival of Northern Thai breast cancer patients in both staging systems was comparable to the Southeast Asian cohorts, but it was lower than that of high resource countries cohorts. This discrepancy in cancer survival confirms the importance of access to screening program and therapeutic agents and facilities for breast cancer patients in Northern Thailand.
